# Short-term effects of diaphragmatic myofascial release on pain, mobility, and disability in chronic nonspecific low back pain: a randomized sham-controlled trial

**DOI:** 10.1038/s41598-026-56878-8

**Published:** 2026-06-05

**Authors:** Soheil Hejazi Yekta, Khadijeh Otadi, Kazem Malmir, Sara Fereydounnia

**Affiliations:** Physiotherapy Department, School of Rehabilitation, Tehran University of Medical Sciences, P. O. Box: 113635–1683, Tehran, Iran

**Keywords:** Chronic nonspecific low back pain, Diaphragmatic myofascial release, Disability, Balance, Pain, Diseases, Health care, Medical research, Neurology, Neuroscience

## Abstract

Chronic nonspecific low back pain (CNSLBP) is a common condition often associated with impaired diaphragmatic and trunk function. The therapeutic effect of diaphragmatic myofascial release (DMR) in this population requires clarification. The aim of this study was to investigate the short-term effects of DMR on pain, mobility, balance, and disability in CNSLBP. In a randomized sham-controlled trial, twenty-four adults with CNSLBP were randomly assigned to receive three sessions of either DMR or a sham intervention. Both groups also received standardized transcutaneous electrical nerve stimulation (TENS). The primary outcome was pain intensity measured on a Visual Analog Scale (VAS). Secondary outcomes included lumbopelvic mobility (fingertip-to-floor test), static and dynamic balance (single-leg stance and functional reach tests), disability (Roland-Morris Disability Questionnaire, RMDQ), and chest expansion, were measured at baseline, after the third intervention session, and at one-week follow-up. A significant group × time interaction was detected for VAS (*p* < 0.001, η²ₚ = 0.33); the intervention group showed a significantly larger pain reduction than the sham group. Fingertip-to-floor also showed a significant group × time interaction (*p* = 0.04, η²ₚ = 0.13). Post-hoc tests showed a non-significant trend towards improvement in the intervention group from baseline to post-intervention (MD = 2.54 cm, *p* = 0.07) and from baseline to follow-up (MD = 1.93 cm, *p* = 0.08), whereas no such trend was observed in the control group (*p* > 0.05). Single-leg stance tests improved over time in both groups (*p* < 0.01) but there was no significant group × time interaction. No significant group or interaction effects were observed for functional reach or chest expansion. The RMDQ score decreased significantly in both groups, and the between-group comparison for disability change scores was statistically significant (*p* = 0.036), favoring the intervention group. DMR combined with TENS resulted in greater short‑term pain reduction and disability improvement, both exceeding minimal clinically important differences, compared with sham, and a non‑significant trend toward improved lumbar mobility. Static balance improved over time in both groups, with no between‑group differences. These results support DMR as a promising adjunct for managing pain, disability, and potentially balance in CNSLBP, but larger trials are needed to confirm its specific effects.

Trial registration: IRCT20221216056836N1 (IRCT), registered on 28 February 2023.

**Supplementary Information:**

The online version contains supplementary material available at 10.1038/s41598-026-56878-8.

## Introduction

Low back pain (LBP) is a common health problem and one of the most frequent reasons for which people seek medical treatment^1^. Lifetime prevalence reaches 80%, with 1–40% among young athletes^2^. The condition also imposes a substantial socioeconomic burden through direct healthcare expenditures and indirect costs from lost work productivity and long‑term disability. Many adults experience chronic LBP, which often presents with lasting limitations in their daily activities^3^. Chronic nonspecific LBP (CNSLBP) is defined as pain between the lower ribs and the gluteal folds without a specific structural cause^4^. It is particularly challenging to manage. This is because imaging and structural findings often do not explain pain severity or functional limitations^5^, leaving clinicians without reliable biomarkers or targeted therapies. The diagnosis of non‑specific LBP assumes no identifiable nociceptive source or pathoanatomical cause, and routine imaging is discouraged in the absence of red flags^2^. Even when a diagnosis is established, treatment remains challenging: contemporary clinical practice guidelines consistently recommend non-pharmacological therapies such as exercise and spinal manipulation, but evidence for many adjunctive manual techniques is limited and recommendations vary widely across countries^6,7^.

The causes of CNSLBP are complex and not fully understood. Although some studies suggest links with spinal degeneration^8^, similar imaging findings are often seen in people without symptoms^9^, which makes it difficult to attribute pain to structural changes. Posture, movement patterns, and mechanical strain show inconsistent links to symptoms^10,11^. These therapeutic uncertainties create a pressing need for novel treatment targets.

Recent attention has shifted toward functional problems in the musculoskeletal system, especially muscle imbalance and reduced spinal stability^12,13^. The diaphragm is not only a respiratory muscle but also a crucial stabilizer of the lumbar spine^14^. In people with chronic LBP, diaphragm function is often impaired: its ability to regulate intra-abdominal pressure and coordinate with other trunk muscles is reduced, which can lead to compensatory overactivation of superficial paraspinal muscles, faulty movement strategies, and sustained nociceptive input that contributes to pain chronicity^13,14^. Manual therapy techniques, including myofascial release, have been proposed to address these dysfunctions. In individuals with chronic LBP, such techniques have led to improvements in trunk mobility, dynamic standing balance, pain intensity, and self‑reported function^14,15^.

Several studies have reported benefits from diaphragm-focused interventions for pain and mobility. For example, one study found that osteopathic techniques directed at the diaphragm produced greater reductions in pain and disability than a sham procedure^16^. Another study reported that releasing tension in the diaphragm and adjacent muscles improved lumbar flexibility and reduced discomfort in people with chronic LBP^17^. However, the current study is novel in that it specifically examines diaphragmatic myofascial release (DMR) as a focused manual technique targeting only the diaphragm, rather than in combination with iliopsoas release, which was the focus of the referenced study. Furthermore, our study introduced new outcome measures, including static and dynamic balance assessment, lumbopelvic mobility, and LBP–related disability, which were not evaluated in the referenced study. This adds significant value by expanding the understanding of how DMR may influence postural control, respiratory function, and functional disability in patients with CNSLBP. Because the diaphragm is integral to spinal stability and postural control^18^, releasing its fascial restrictions could improve balance, either directly through biomechanical mechanisms or indirectly through pain relief. Previous work has shown that myofascial release applied to the trunk can enhance dynamic standing balance in individuals with chronic LBP^14^, supporting the rationale for including balance and disability as outcomes in the present trial. Transcutaneous electrical nerve stimulation (TENS) was given to both groups for three reasons. First, because both arms received the same electrical stimulation, any improvement in the DMR group beyond the sham group could be attributed specifically to the active myofascial release technique, rather than to extra touch, attention, or electro‑analgesia. Second, TENS helped keep pain levels stable during assessments, reducing variability that could have obscured a treatment effect in this small trial. Third, the design reflects a practical clinical decision—whether adding a few minutes of DMR to a standard TENS session yields extra benefit—which is directly relevant to everyday physiotherapy practice.

Despite these positive findings on pain and mobility, critical evidence gaps persist that limit clinical use of DMR. Specifically, its influence on balance, chest expansion, and daily function remains unclear. Furthermore, its efficacy as an adjunct to common therapies like TENS is not yet established. Addressing these gaps is essential—not only to determine DMR’s comprehensive therapeutic potential in CNSLBP management, but also to inform evidence-based clinical guidelines. Thus, there is a strong need for rigorous, sham-controlled trials evaluating DMR combined with TENS across multiple outcomes including pain, mobility, balance, and disability. This randomized sham‑controlled trial was designed as a short‑term proof‑of‑concept investigation, evaluating the immediate and one‑week effects of DMR on these outcomes. The short‑term focus allows the generation of effect‑size estimates and feasibility data to inform the design of future definitive trials with longer follow‑up periods.

## Method

### Participants

A total of 24 patients with CNSLBP were enrolled and randomly assigned to either the intervention group or the control group, with 12 participants in each group. Randomization was performed using block randomization with a block size of 4 and an allocation ratio of 1:1, generated by Random Allocation Software. The sample size was determined a priori using G*Power software (version 3.1) based on preliminary data for chest expansion at the xiphoid process (pre-treatment mean = 1.75 cm, SD = 0.64; post-treatment mean = 2.6 cm, SD = 0.81) and pain intensity measured on a Visual Analog Scale (VAS) (pre-treatment mean = 4.2 cm, SD = 0.19; post-treatment mean = 3.9 cm, SD = 0.25). An independent samples t-test was specified with an alpha of 0.05 and 80% power. The analysis yielded an effect size of 1.16 for chest expansion, indicating a requirement of 20 participants (10 per group), and an effect size of 1.351 for VAS, indicating 16 participants (8 per group). The larger sample size (*n* = 20) was selected to ensure adequate power. To account for potential attrition, the final allocation was increased to 12 participants per group (24 total).

Participants were male and female individuals aged 18–40 years. The diagnosis was confirmed by an orthopedic specialist. Participants presented with pain lasting more than three months, localized between the lower ribs and the gluteal folds without radiation below the gluteal fold, and a baseline pain intensity rated between 3 and 6 on the VAS. This range was selected to target individuals with moderate pain intensity, thereby minimizing floor and ceiling effects and increasing the likelihood of detecting a treatment-related change in this study. It also reduced the risk of including participants whose pain severity might require immediate alternative medical management. Individuals with neurological signs, spinal fractures, active infection, or prior spine surgery were excluded. To minimize carryover effects from prior interventions, participants who had received structured physiotherapy within the preceding three months were excluded.

### Procedures

This was a randomized, sham-controlled, parallel-group trial. The study protocol was registered in the Iranian Registry of Clinical Trials (IRCT20221216056836N1, https://irct.behdasht.gov.ir/trial/67486, registered on 28 February 2023) and approved by the Ethics Committee of the School of Rehabilitation, Tehran University of Medical Sciences (IR.TUMS.FNM.REC.2022.12.11). Recruitment occurred from April to July 2023; all assessments and treatments were performed at the Rehabilitation Research Laboratory, School of Rehabilitation, TUMS. Participants and the outcome assessor were blinded to group allocation throughout the trial. Group allocation was concealed using sequentially numbered, opaque, sealed envelopes.

Prior to participation, written informed consent was provided by all individuals, which included permission for the use of their data and images in publications. The allocation sequence was prepared by an independent researcher not involved in the study’s conduct. Group assignments were placed in sequentially numbered, opaque, sealed envelopes, which were also prepared by an independent researcher. These envelopes were opened only after baseline assessments were completed, ensuring allocation concealment. Treatments were provided by one licensed physiotherapist. This physiotherapist did not perform outcome assessments. A second physiotherapist, blinded to group assignment, conducted all outcome measurements. These measurements were taken at baseline, after the third intervention session, and one week after the final intervention. The short-term treatment and assessment protocol was specifically chosen to align with the study’s primary objective as a proof-of-concept efficacy trial. A three‑session protocol was adopted because a single session of diaphragmatic myofascial release has been shown to produce immediate improvements in pain and mobility^19^, and three sessions allowed us to assess whether these improvements are maintained over a short period. The one‑week follow‑up was chosen to evaluate the durability of any post‑intervention gains without requiring an extended period of restricted activity. This design provides preliminary data on immediate and short‑term effects to inform sample size calculations and protocol refinement for future definitive trials, while acknowledging that longer follow‑up is required to assess sustained clinical benefit.

### Interventions

**Fig. 1:**
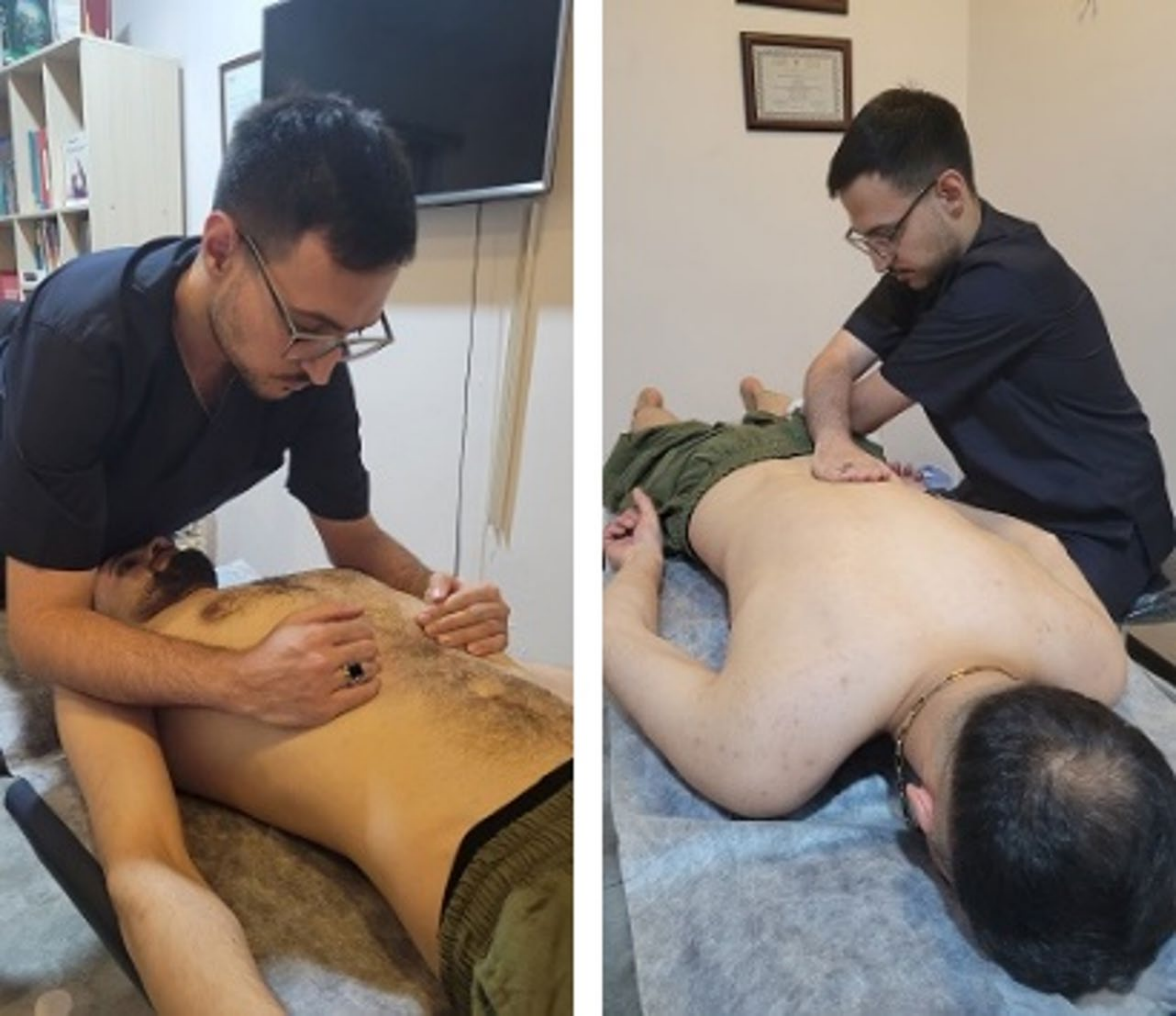
Application of diaphragmatic myofascial release techniques. (Left) Supine approach targeting the costal arch; (Right) Prone technique addressing posterior fascial restrictions.

Participants in the intervention group received DMR performed by a licensed physiotherapist using two manual techniques. In the first (supine) technique, the participant lay on their back while the therapist stood at the head of the treatment table and placed the hypothenar region and the medial three fingers on the cartilaginous portions of ribs 7–10 bilaterally. During inhalation, the therapist applied a gentle cranial and lateral mobilizing force to help the ribs elevate, and during exhalation, the therapist provided resistance to rib motion (Fig. 1). This cycle was repeated for two sets of 10 repetitions^20^.

In the second (prone) technique, the participant lay face down. The therapist placed one hand on the posterior lower ribs and the other hand on the popliteal fossa of the same-side lower limb. During exhalation, the therapist applied a gentle separating force between the hands (without sliding the hands on the skin) and released the force during inhalation (Fig. 1); this was performed for one minute on each side^19^.

The sham group received identical hand placements and duration of contact but no mobilizing or resistive forces, so that the tactile contact of therapist’s touch was matched without delivering the active techniques. In addition, both groups received a standardized 20-minute session of conventional TENS to the lumbar region (100 Hz frequency, 200 µs pulse duration). Intensity was increased until the participant reported a strong but comfortable tingling sensation without visible muscle contraction (sensory threshold), and was readjusted as needed to maintain this sensation throughout the 20-minute session. Two electrodes were placed horizontally at the L4-L5 interspace, 2–3 cm lateral to the midline bilaterally, to target the erector spinae muscles^21^. All interventions were delivered in three sessions. All participants in the sham group were offered and received the full, active intervention protocol immediately after the completion of their final follow-up assessment. To prevent information contamination between groups, sessions were held separately at separate times with uniform participant instructions. In addition, participants received identical preliminary instructions. Participants were asked not to receive any other physiotherapy or medical treatments during the study period, and the intervention was stopped for any participant who reported increased pain during or between sessions.

### Outcome measures

The primary outcome of this study was pain intensity. Secondary outcomes included lumbopelvic mobility, static and dynamic balance, disability, and chest expansion. All outcomes (except for the LBP–related disability) were measured at baseline, immediately after the third intervention session, and at a one-week follow-up. All outcome measurements were obtained by a physiotherapist who was blinded to group allocation.

Pain intensity was measured using a 10-cm paper VAS on which 0 = no pain and 10 = worst imaginable pain; participants indicated their current pain by marking the line. Pain was recorded at all three assessment time points^22^. It demonstrates moderate to good reliability, with intraclass correlation coefficient (ICC) values of 0.75–0.94^23^ and an Minimal Clinically Important Difference (MCID) of approximately 1.5 cm for CNSLBP^24^.

LBP–related disability was measured with the Roland-Morris Disability Questionnaire (RMDQ), a validated 24-item yes/no questionnaire in which each “yes” scores one point (total range 0–24), with higher scores showing greater disability. The RMDQ was completed at baseline and at the one-week follow-up, and we used the validated version^25^. The RMDQ has demonstrated good internal consistency, with a Cronbach’s alpha of 0.83, and excellent test-retest reliability, with an ICC of 0.86 in chronic LBP^26^.

Static balance was assessed with a single-leg stance test. Participants stood on one leg with arms crossed over the chest and performed three trials each with eyes open and eyes closed. A stopwatch recorded how long they maintained the stance (maximum 45 s), and the mean of the three trials was used for each vision condition. This test was performed at all three time points^27^. The single limb stance test demonstrates excellent inter-rater reliability, with kappa values ranging from 0.88 to 1.0^28^.

**Fig. 2:**
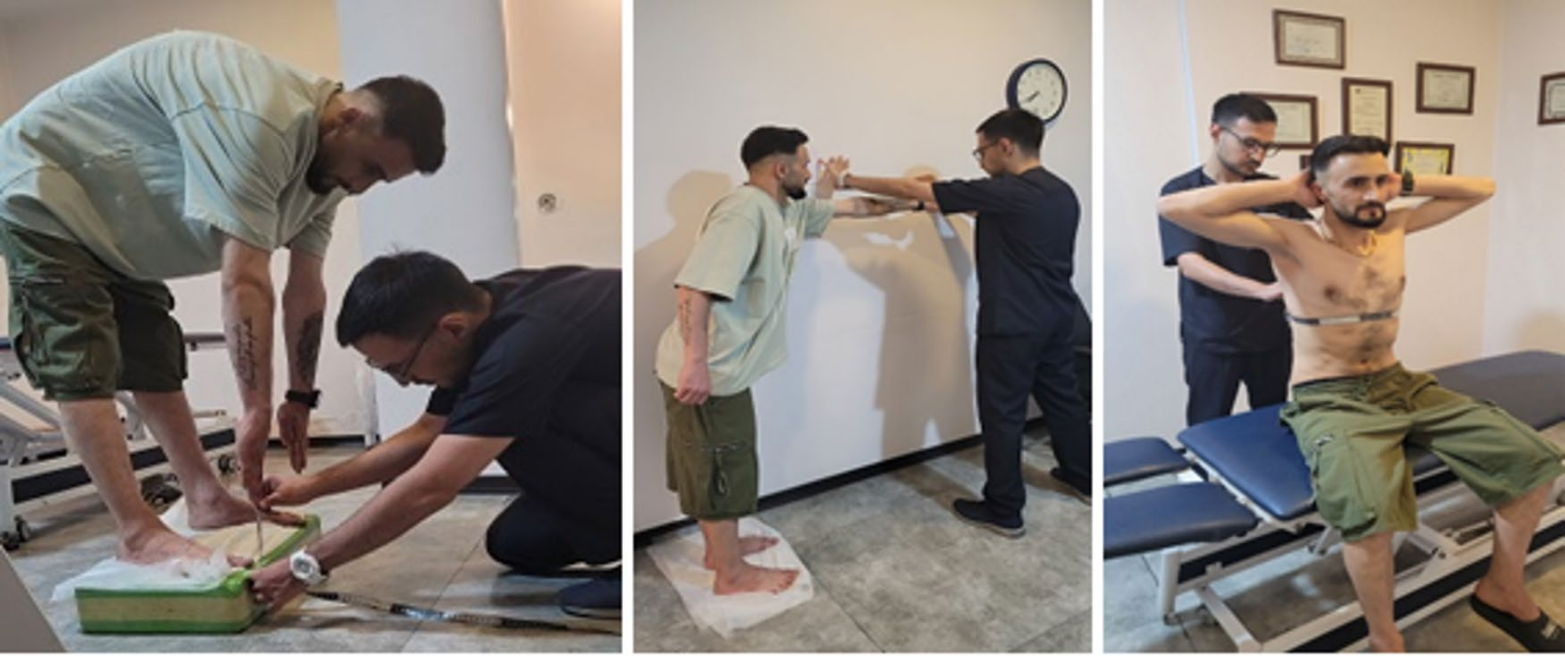
(Left) Fingertip-to-Floor distance during maximal forward flexion; (Middle) Functional Reach Test demonstrating maximum forward reach distance; (Right) Chest expansion measurement at the xiphoid level during maximal inhalation and exhalation.

Dynamic balance was measured using the Functional Reach Test (Fig. 2). Participants stood beside a wall with feet behind a marked line and reached forward as far as possible without stepping or losing balance; the distance between the start and maximal reach was measured with a tape measure. This test was repeated at every assessment^29^. It has shown moderate to good reliability, with ICC values ranging from 0.74 to 0.98^30^.

Lumbopelvic mobility was evaluated by the Fingertip-to-Floor distance test (Fig. 2). From a standing position with knees straight, participants bent forward and the distance from the tip of the third finger to the floor was measured with a tape measure. Values were recorded in centimeters, with 0 cm representing the floor level. A negative value indicates the finger reached below the level of the feet, with more negative values indicating greater forward flexion. This measure was taken at each assessment visit^31^. Excellent inter- and intra-observer reliability has been reported for this outcome (ICC = 0.99)^32^.

Chest expansion was measured with the participant seated and a tape measure placed at the level of the xiphoid process (Fig. 2). Participants performed a maximal inhalation followed by a maximal exhalation, and the difference in thoracic circumference was recorded as the chest expansion value^19^. Chest expansion was measured at each time point. This measure has shown good reliability^33^.

### Statistical analysis

Statistical analyses were conducted using SPSS version 25 under an intention-to-treat approach, in which all randomized participants were analyzed in their originally assigned groups. Normality was tested using the Shapiro–Wilk test. For variables that met parametric assumptions, a two-way repeated-measures ANOVA (group × time) was used to examine main effects and the interaction. When sphericity was violated, Greenhouse–Geisser corrections were applied. Bonferroni post hoc tests were used for pairwise comparisons. For variables that did not meet parametric assumptions, the Friedman test was used for within-group comparisons. The Roland-Morris Disability Questionnaire was analyzed with the Wilcoxon signed-rank test for within-group changes and the Mann–Whitney U test for between-group differences in change scores. Baseline demographic differences were assessed with independent-samples t-tests or Mann–Whitney U tests, as appropriate. All tests were two-tailed and a p-value < 0.05 was considered statistically significant. The strength of the effect size (η²p) was categorized as small (0.01), moderate (0.06), or large (0.14)^34^. For non-parametric analyses, the effect magnitude was expressed using the correlation metric “r”, obtained through the transformation of the test statistic Z by the inverse of the sample size’s square root.

## Results

**Fig. 3:**
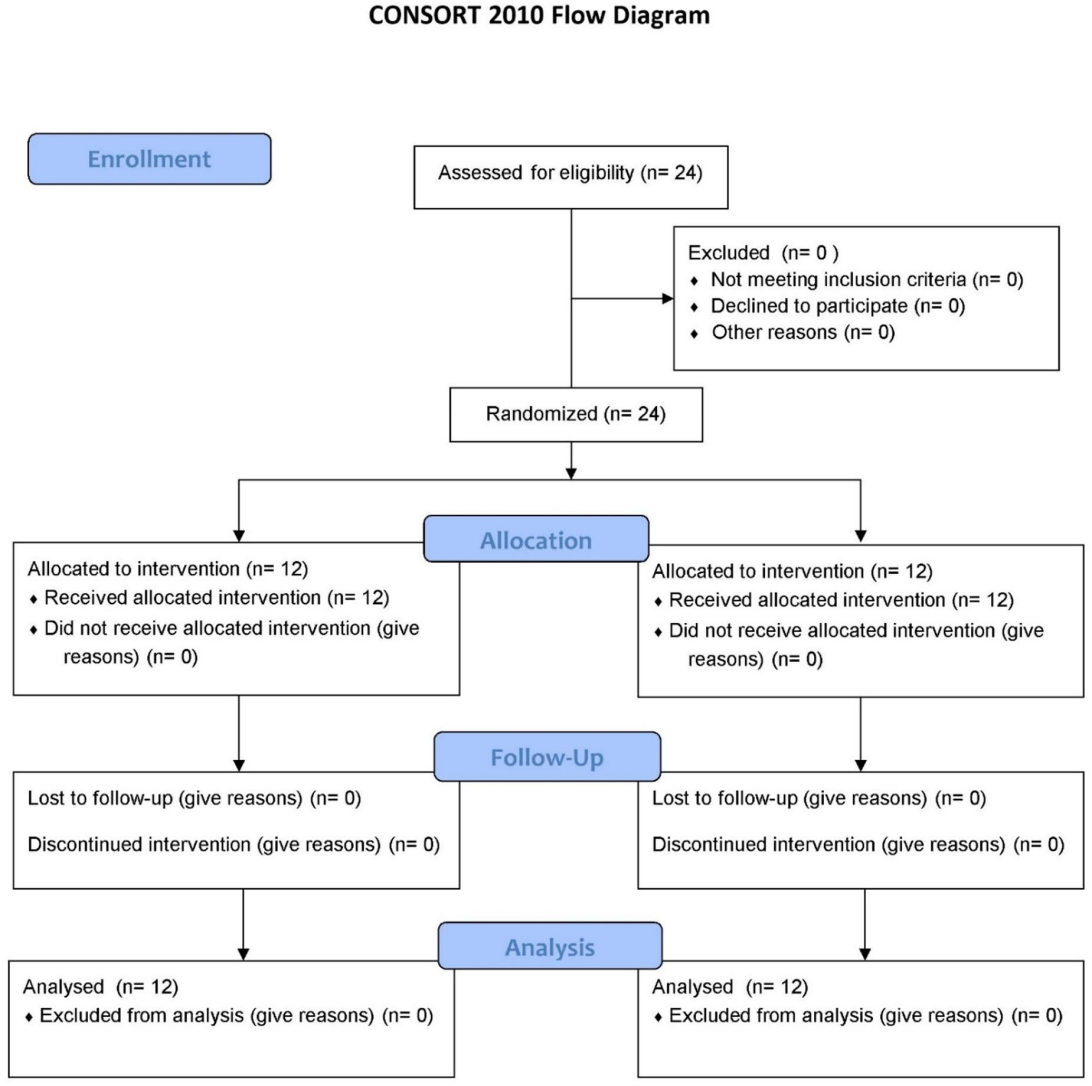
CONSORT flow diagram of the study.

All 24 randomized participants (12 per group) completed the study (Fig. 3). Adverse events were monitored at each visit. All participants in both groups were directly questioned about any new or worsening pain, discomfort, or other complaints beyond their baseline LBP symptoms. No adverse events related to the study procedures were reported. Baseline demographic and clinical characteristics are shown in Table 1. Groups did not differ significantly at baseline on age, height, weight, or body mass index (BMI) (*p* > 0.05).

**Table 1:** Participants’ characteristics.

Variable	Control (*n* = 12)	Intervention (*n* = 12)	*p*-value
Age (year)	29.33 ± 5.19	30.41 ± 6.28	0.65
Height (cm)	170.85 ± 6.36	174.95 ± 6.55	0.13
Weight (kg)	70.86 ± 9.06	78.97 ± 11.32	0.07
BMI (kg/m^2^)	24.37 ± 3.40	25.63 ± 3.71	0.39

**Abbreviation**: BMI, body mass index.

Group means and standard deviations for each outcome at baseline, after the third session, and at one-week follow-up are presented in Table 2.

**Table 2:** Group means and standard deviations for all outcomes at each time point (*n* = 12 per group).

Outcome measure	Group	Baseline	After third-session	Follow-up
VAS (cm)	Control	4.08 ± 0.67	3.76 ± 0.84	3.59 ± 0.96
	Intervention	4.17 ± 0.57	2.36 ± 0.80	2.14 ± 1.07
Chest expansion (cm)	Control	3.67 ± 1.34	3.78 ± 1.50	4.04 ± 1.18
	Intervention	4.24 ± 2.03	4.78 ± 2.04	4.44 ± 1.69
Fingertip-to-floor (cm)	Control	-1.28 ± 10.98	-1.18 ± 11.15	-2.12 ± 11.66
	Intervention	-2.77 ± 8.19	-5.31 ± 9.06	-4.70 ± 8.93
Functional Reach (cm)	Control	33.82 ± 3.75	34.13 ± 5.34	34.06 ± 5.42
	Intervention	33.75 ± 5.59	35.44 ± 6.12	34.62 ± 5.43
Static balance (CE) (s)	Control	7.78 ± 8.31	10.28 ± 11.13	8.47 ± 7.65
	Intervention	5.18 ± 4.24	8.00 ± 5.34	6.76 ± 3.30
Static balance (OE) (s)	Control	32.41 ± 11.39	38.33 ± 9.37	37.95 ± 9.53
	Intervention	34.11 ± 5.16	39.69 ± 5.88	42.23 ± 3.89
RMDQ (0–24)	Control	6.75 ± 5.42	NA	6.00 ± 4.93
	Intervention	6.17 ± 3.15	NA	3.58 ± 1.80

**Abbreviations**: VAS, visual analogue scale; CE, closed eyes; OE, open eyes; RMDQ, Roland-Morris Disability Questionnaire; NA, not assessed. Greater forward flexion in the Fingertip-to-floor test is represented by more negative values.

**Fig. 4:**
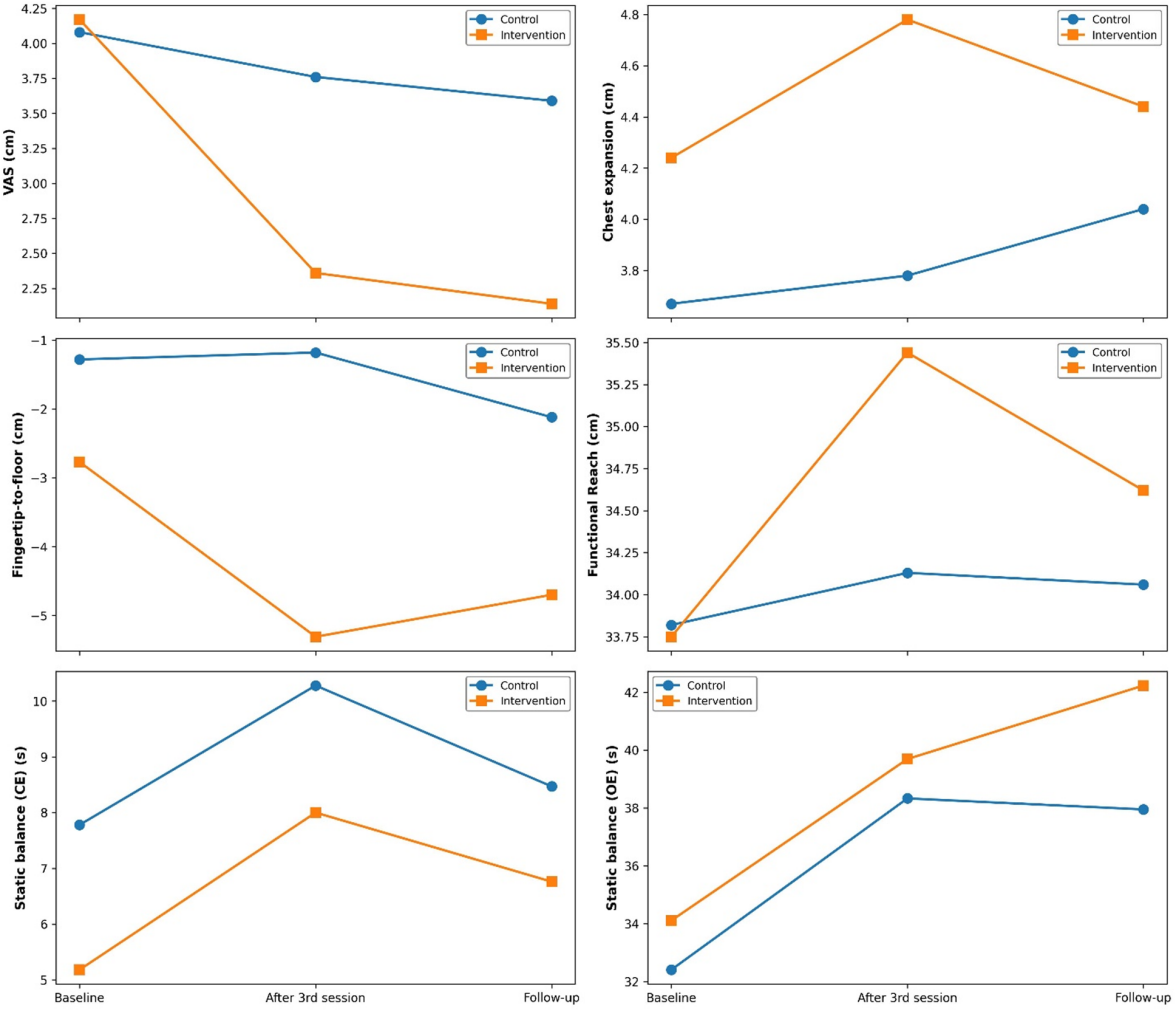
Trends in mean values for the intervention and control groups across each outcome measure at baseline, after third session, and one-week follow-up.

The results of the two-way repeated-measures ANOVA for main and interaction effects are summarized in Table 3, and Bonferroni-adjusted pairwise comparisons are detailed in Table 4. Trends in group means across the three assessment points are illustrated in Fig. 4.

### Pain intensity

A significant group × time interaction with large effect size was observed for VAS (*p* < 0.001, η²ₚ = 0.33), indicating that the scores evolved differently over time between the groups. Post-hoc analysis showed that the intervention group experienced a significant and rapid reduction in pain from baseline to post-intervention (mean difference [MD] = 1.81, 95% CI [1.17 to 2.44], *p* < 0.001), which was sustained at the one-week follow-up (MD = 2.03, 95% CI [1.15 to 2.92], *p* < 0.001). The confidence intervals for these changes do not cross zero, which underscores the robustness of the finding. In contrast, the control group showed no statistically significant reduction from baseline to follow-up (MD = 0.49, 95% CI [-0.14 to 1.12], *p* = 0.14).

### Chest expansion

Chest expansion showed no significant group × time interaction (*p* = 0.17, η²ₚ = 0.07), nor were there significant main effects for group (*p* = 0.35, η²ₚ = 0.03) or time (*p* = 0.15, η²ₚ = 0.10). These results indicate that neither the intervention nor the passage of time significantly impacted this outcome. This suggests that chest expansion remained relatively stable in both groups over the study period, implying that the intervention did not influence this specific measure of mobility or function.

### Lumbopelvic mobility

The fingertip-to-floor test showed a significant group × time interaction (*p* = 0.04, η²ₚ = 0.13). This indicates a different pattern of change between the groups. Post-hoc pairwise comparisons with Bonferroni correction were performed. The intervention group showed positive trends from baseline to post-intervention (MD = 2.54 cm, 95% CI [-0.21 to 5.29], *p* = 0.07). Positive trends were also seen from baseline to follow-up (MD = 1.93 cm, 95% CI [-0.21 to 4.07], *p* = 0.08). However, these changes were not statistically significant because the confidence intervals included zero. No significant within-group changes were observed in the control group at any time point.

### Static balance

For the eyes closed condition, a significant main effect of time with a large effect size was found (*p* = 0.005, η²ₚ = 0.21), but no significant group × time interaction (*p* = 0.80). Post-hoc comparisons showed that the intervention group significantly improved from baseline to post-intervention (MD = -2.82, 95% CI [-5.38 to -0.25], *p* < 0.001), with no retention at follow-up (MD = -1.57, 95% CI [-3.39 to 0.24], *p* = 0.10). The control group showed no significant changes at any time point (baseline to post-intervention: MD = -2.39, 95% CI [-5.65 to 0.86], *p* = 0.19; baseline to follow-up: MD = -0.59, 95% CI [-3.70 to 2.52], *p* = 1.00).

For the eyes open condition, a significant main effect of time with a very large effect size was observed (*p* = 0.001, η²ₚ = 0.44). There was no significant group × time interaction (*p* = 0.41). Post-hoc analysis showed the intervention group significantly improved from baseline to post-intervention (MD = -5.58, 95% CI [-9.90 to -1.25], *p* = 0.008). This improvement was further maintained at follow-up (MD = -8.12, 95% CI [-11.35 to -4.88], *p* = 0.001). In contrast, the control group showed no significant within-group improvements in static balance for either condition (*p* > 0.05).

### Dynamic balance (functional reach)

Functional reach showed no significant group × time interaction (*p* = 0.76, η²ₚ = 0.004), main effect of group (*p* = 0.47, η²ₚ = 0.03), or main effect of time (*p* = 0.69, η²ₚ = 0.01), indicating that neither the intervention nor time alone had a significant impact on dynamic balance. The confidence intervals for all comparisons crossed zero, and no pairwise comparisons reached statistical significance, suggesting that there was no significant change in functional reach over time in either group. Both groups remained relatively stable in their functional reach measurements, indicating that the intervention did not influence this particular measure of balance or mobility.

### Self-reported disability

Within-group analysis using the Wilcoxon signed-rank test showed a significant reduction in RMDQ scores from baseline to one-week follow-up in the intervention group (Z = − 2.820, *p* = 0.005, *r* = 0.81). The sham group also demonstrated a significant improvement (Z = − 1.983, *p* = 0.047, *r* = 0.57). The Mann-Whitney U test comparing the change scores (follow-up minus baseline) between groups revealed a significantly greater improvement in the intervention group (mean rank = 9.54) than in the sham group (mean rank = 15.46; U = 36.5, *p* = 0.036, *r* = 0.43). These findings indicate that the addition of DMR produced a superior reduction in LBP-related disability compared to sham intervention, with a moderate-to-large effect size.

**Table 3:** Results of two-way repeated-measures ANOVA for main and interaction effects across all outcome measures (*n* = 12 per group).

	F-ratio				*p-value*				η²ₚ		
	Group	Time	Interaction		Group	Time	Interaction		Group	Time	Interaction
VAS	12.01	25.95	10.81		**0.002**	**< 0.001**	**< 0.001**		0.35	0.54	0.33
Chest-expansion	0.90	2.46	1.89		0.35	0.15	0.17		0.03	0.10	0.07
Fingertip-to-floor test	0.45	4.30	3.30		0.50	**0.02**	**0.04**		0.02	0.16	0.13
Functional Reach	0.09	0.76	0.36		0.76	0.47	0.69		0.004	0.03	0.01
Static balance (CE)	0.63	6.10	0.21		0.43	**0.005**	0.80		0.02	0.21	0.01
Static balance (OE)	0.63	17.46	0.82		0.43	**0.001**	0.41		0.03	0.44	0.03

**Abbreviations**: VAS, visual analogue scale; CE, closed eye; OE, open eye; Bold values indicate statistically significant results (*p* < 0.05).

**Table 4:** Bonferroni-adjusted within-group pairwise comparisons for all outcome measures across the three time points (*n* = 12 per group).

Variable	Group	Pairwise comparison	Mean difference (adjusted) (95% CI)	*p*-value
VAS	Intervention	Baseline – Third session	1.81 [1.17 to 2.44]	**< 0.001**
		Baseline – Follow-up	2.03 [1.15 to 2.92]	**< 0.001**
		Third session – Follow-up	0.23 [-0.49 to 0.95]	1.00
	Control	Baseline – Third session	0.32 [-0.35 to 0.98]	0.20
		Baseline – Follow-up	0.49 [-0.14 to 1.12]	0.14
		Third session – Follow-up	0.17 [-0.74 to 1.09]	0.50
Fingertip-to-floor test	Intervention	Baseline – Third session	2.54 [-0.21 to 5.29]	0.07
		Baseline – Follow-up	1.93 [-0.21 to 4.07]	0.08
		Third session – Follow-up	-0.61 [-2.19 to 0.97]	0.90
	Control	Baseline – Third session	-0.10 [-1.70 to 1.50]	0.80
		Baseline – Follow-up	0.84 [-1.41 to 3.09]	0.31
		Third session – Follow-up	0.94 [-0.84 to 2.72]	0.16
Static balance test (CE)	Intervention	Baseline – Third session	-2.82 [-5.38 to -0.25]	**< 0.001**
		Baseline – Follow-up	-1.57 [-3.39 to 0.24]	0.10
		Third session – Follow-up	1.25 [-1.45 to 3.94]	0.15
	Control	Baseline – Third session	-2.39 [-5.65 to 0.86]	0.19
		Baseline – Follow-up	-0.59 [-3.70 to 2.52]	1.00
		Third session – Follow-up	1.80 [-2.22 to 5.83]	0.70
Static balance test (OE)	Intervention	Baseline – Third session	-5.58 [-9.90 to -1.25]	**0.008**
		Baseline – Follow-up	-8.12 [-11.35 to -4.88]	**0.001**
		Third session – Follow-up	-2.54 [-5.94 to 0.86]	0.41
	Control	Baseline – Third session	-5.92 [-12.38 to 0.53]	0.07
		Baseline – Follow-up	-5.54 [-13.05 to 1.96]	0.18
		Third session – Follow-up	0.38 [-2.55 to 3.32]	1.00

**Abbreviations**: VAS, visual analogue scale; CE, closed eyes; OE, open eyes; Bold values indicate statistically significant differences (*p* < 0.05).

## Discussion

This randomized sham‑controlled trial demonstrated that adding three sessions of active DMR to standardized TENS was significantly more effective than adding sham manual therapy to the same TENS regimen in producing short‑term reductions in pain, indicating that the between‑group differences are attributable to the specific effects of the myofascial release technique rather than to TENS or therapist contact alone. The data also showed a trend towards improved lumbopelvic mobility. Static balance improved over time in both groups, with no significant between-group difference. Self-reported disability improved significantly within the intervention group to a clinically meaningful degree; the between-group comparison for disability change scores was statistically significant, favoring the intervention group.

### Pain intensity

The present randomized trial demonstrated that three sessions of DMR combined with standardized TENS produced clinically and statistically meaningful reductions in pain intensity. The intervention group experienced a mean VAS reduction of 1.80 cm from baseline to the third session and 2.03 cm from baseline to one-week follow-up, both exceeding the commonly cited MCID of about 1.5 cm for CNSLBP^24^. These short-term changes, from a brief 3-session intervention, provide preliminary evidence of clinical relevance. In addition, pain decreased earlier in the DMR group than in the control group.

Our pain results are consistent with prior reports that diaphragm-directed manual techniques and myofascial interventions can reduce pain and improve function in LBP populations^15,17^. The substantial between‑group difference in pain reduction is noteworthy given the brief intervention period of only three sessions. It suggests that adding active DMR to TENS produces a marked short‑term modulatory effect that is not achieved by TENS and sham touch alone. This incremental benefit may be driven by combined mechanical and neurophysiological mechanisms triggered by the active myofascial release.

Several potential hypothesized mechanisms may explain the observed effects. First, myofascial release may reduce local fascial stiffness and adhesions, improving tissue glide and decreasing mechanical nociceptive input. Second, diaphragmatic techniques may improve respiratory-motor coordination and intra-abdominal pressure regulation, which could enhance lumbopelvic stability and reduce protective muscle guarding. Third, although not directly studied for DMR, manual therapy techniques may have been associated with autonomic nervous system modulation, including increased parasympathetic tone, which may contribute to pain reduction^35^. Finally, nonspecific effects (therapist’s touch, expectation, and the concurrent TENS provided to both groups) likely contributed to improvements in both arms, accounting for some within-group gains in the control group.

### Lumbopelvic mobility and disability

In support of the regional interdependence model, the pattern of change in lumbopelvic mobility over time differed between the groups. The intervention group demonstrated mean improvements of 1.93–2.54 cm (comparing with 0.83 cm improvement in the control group), which suggests a tendency toward enhanced mobility specifically attributable to the active DMR component. Although these improvements did not reach the MCID threshold of approximately 4.5 cm^31^, they offer a preliminary indication of potential short-term clinical relevance from the 3-session protocol. Given the nature of these findings, further investigation with a larger trial is warranted to clarify the intervention’s effect. The improvement observed during the first three sessions in both groups may be due to neurophysiological effects, as myofascial release stimulates receptors distributed in the myofascial membrane, which can lead to increased body flexibility and enhanced trunk mobility^36^. However, the initial improvement was not maintained after one week of follow-up, possibly due to the temporary nature of these neurophysiological responses and the lack of sustained physiological changes.

In the intervention group, RMDQ scores showed a notable reduction, decreasing from 6.17 ± 3.15 at baseline to 3.58 ± 1.80 at one‑week follow‑up. The mean improvement of 2.59 points exceeded the MCID threshold of 2–3 points^37^, indicating clinically meaningful short‑term improvement after only three sessions. The control group also demonstrated some improvement, though to a lesser degree. The significant improvement observed in the sham group warrants consideration. Therapeutic touch alone, even without mobilizing forces, combined with the analgesic effects of TENS, may produce beneficial effects through attentional mechanisms, expectation, and stimulation of cutaneous afferents. This highlights the challenge of designing truly inert sham controls for manual therapy research. When directly comparing the amount of change between groups, the intervention group showed a greater reduction in disability than the control group. These findings suggest that the addition of active DMR to a background of real TENS provides an added benefit for reducing LBP‑related disability beyond what TENS and sham touch alone can achieve; by achieving superior pain relief, the intervention group likely experienced greater ease in daily activities, leading to a larger drop in perceived disability. Replication in larger, longer‑term trials is warranted to confirm the durability of these disability improvements.

### Chest wall expansion

Regarding chest expansion, scores followed a similar pattern over time in both groups, so the added diaphragmatic release brought no extra benefit over the sham touch. Measured chest expansion did not show a clear change from baseline at either the post-third session or one-week follow-up assessments. Several factors may explain this finding: the brief intervention duration, the inherent measurement variability of tape assessments, or the possibility that tactile manual release alone is insufficient to modify thoracic circumference in three sessions without integrated breathing retraining. Supporting this, studies combining manual techniques with respiratory training or using imaging to measure diaphragmatic excursion have reported internal physiological changes not consistently captured by external circumference measures^17,38^.

### Static and dynamic balance

For static and dynamic balance, the pattern of change over time did not show a clear distinction between the intervention and control groups. Static balance, measured both with eyes open and closed, improved over time, with these gains observed within the intervention group itself. Because a similar pattern of improvement was also evident in the control group, the changes cannot be specifically attributed to the DMR technique. This may be due to the general reduction in LBP intensity and improvement in physical function^39^, as well as the effects of tactile contact (sham touch) and TENS, which may produce short-term sensorimotor or attentional effects^36,40^. For dynamic balance, the differences between groups were minimal. Several explanations are plausible:^1^ both groups received tactile contact (sham touch) and TENS, which may produce short-term sensorimotor or attentional effects^36,40^;^2^ three sessions may be insufficient for specific neuromotor re-training needed to translate diaphragmatic mobility gains into measurable postural control improvements; and^3^ single-leg stance and functional reach tests were selected deliberately as simple, functional bedside measures that are recommended for balance screening in LBP populations and have previously shown responsiveness to myofascial release interventions. For instance, Lee et al.^14^ reported that dynamic myofascial release applied to the trunk led to measurable improvements in the functional reach test and single-leg stance time in individuals with CNSLBP, demonstrating that these clinical tools possess adequate sensitivity to capture treatment-related balance changes. The fact that our study found no difference between the DMR and sham groups on these same tests therefore cannot be attributed solely to insufficient measurement sensitivity; rather, it indicates that the addition of isolated diaphragmatic myofascial release to TENS does not produce a meaningful additive improvement in functional balance beyond that afforded by the sham intervention and electrotherapy alone. Therefore, balance gains may require either a longer treatment period, a more global trunk-focused myofascial approach, or the integration of active postural training. Dynamic balance similarly showed small, non-significant group differences, consistent with the idea that active motor training (core activation, task practice) is usually required to change dynamic postural control. This aligns with the understanding that balance function requires the integration of sensory input, central nervous system processing, and neuromuscular activity, and that impaired proprioception and neuromuscular dysfunction in CLBP patients may limit the effectiveness of short-term interventions^41,42^.

### Clinical implications and future directions

Based on these results, DMR can be positioned as a valuable adjunct to TENS for the early management of CNSLBP, with the active release technique providing rapid pain relief and reduction of perceived disability beyond what TENS and sham touch achieve. Clinicians might consider incorporating DMR at the outset of treatment to quickly lower pain levels, creating a window for patient engagement in exercise-based rehabilitation while minimizing protective muscle guarding. Exploratory findings suggest trends toward benefits in lumbopelvic mobility. However, to achieve substantial improvements in objective mobility and balance, DMR should likely be combined with active interventions. These could include core stabilization exercises and structured balance training to build on DMR’s pain-modulating foundation and address persistent functional limitations observed at one-week follow-up.

This study has limitations. Although the sample size was determined by an a priori power analysis, it remains relatively small, which may affect the generalizability of the findings. In addition, the inclusion criteria restricted eligibility to adults aged 18–40 years to minimize age‑related confounding in this proof‑of‑concept trial; consequently, the results may not apply to older adults with CNSLBP, among whom degenerative changes and comorbidities are more common. Other limitations include the short-term follow-up and the lack of objective measures of diaphragmatic function (e.g., ultrasonography). Future research should employ larger sample sizes and longer follow-up periods. A critical next step would be to use a factorial design (e.g., DMR vs. sham × with vs. without exercise) to test whether DMR acts as a primer to enhance the effects of active rehabilitation. Incorporating objective physiological measures would also provide crucial insights into the underlying mechanisms of DMR. The absence of RMDQ as an inclusion criterion is another limitation of this study. Future research should incorporate it to refine participant selection. This study measured chest expansion only at the xiphoid level. While this provides an index of general thoracic mobility, it does not capture potential differential changes at the axillary or abdominal levels. Given the diaphragm-focused nature of the intervention, future studies should include abdominal excursion or lower rib cage mobility measures to more precisely evaluate the intervention’s effects on respiratory mechanics. In addition, restricting the VAS inclusion range to 3–6 may limit the applicability of the findings to patients with milder or more severe pain.

## Conclusion

In conclusion, adding a brief intervention of diaphragmatic myofascial release to standardized TENS produced clinically meaningful acute, short‑term reductions in pain and self‑reported disability over and above those seen with TENS and sham manual therapy in patients with CNSLBP, with the disability improvement being meaningfully greater than that observed in the sham group. Trends suggested potential acute benefits in lumbopelvic mobility, but these showed limited retention at one-week follow-up. Static balance improved over time, but not differentially between groups, with transient gains. The intervention did not differentially affect chest expansion or balance compared to a sham control. DMR appears to be a promising adjunct to conventional care, particularly for initial, acute pain modulation and superior reduction of perceived disability. However, larger trials are needed to definitively establish its sustained impact on objective mobility measures and to fully establish its role in clinical practice, potentially in combination with active exercise.

## Supplementary Information

Below is the link to the electronic supplementary material.

Supplementary Material 1

## Data Availability

The data that support the findings of this study are available on request from the corresponding author.

## Abbreviations

CNSLBP: Chronic non-specific low back pain
DMR: Diaphragmatic myofascial release
VAS: Visual analogue scale
RMDQ: Roland–Morris Disability Questionnaire
CE: Closed eye
OE: Open eye
BMI: Body mass index
MCID: Minimal Clinically Important Difference
TENS: Transcutaneous Electrical Nerve Stimulation
